# Association between Breast Milk Mineral Content and Maternal Adherence to Healthy Dietary Patterns in Spain: A Transversal Study

**DOI:** 10.3390/foods9050659

**Published:** 2020-05-20

**Authors:** Cristina Sánchez, Cristina Fente, Rocío Barreiro, Olga López-Racamonde, Alberto Cepeda, Patricia Regal

**Affiliations:** 1 Pharmacy Faculty, Campus Monteprincipe, San Pablo-CEU University, 28668 Madrid, Spain; c.sanchez127@usp.ceu.es; 2 Department of Analytical Chemistry, Nutrition and Bromatology, Santiago de Compostela University, 27002 Lugo, Spain; rocio.barreiro@usc.es (R.B.); Olga.Lopez.Racamonde@sergas.es (O.L.-R.); alberto.cepeda@usc.es (A.C.); patricia.regal@usc.es (P.R.); 3 Midwifery Service at San Roque Health Center, SERGAS (Servizo Galego de Saúde), 27002 Lugo, Spain

**Keywords:** breast milk, ICP-MS, minerals, lactating time, Mediterranean Diet, Atlantic Diet

## Abstract

The composition of breast milk is influenced by many factors, some of which dependent on the mother and others on the child. Changes in lactation and other factors depending on the mother’s physiology and anthropometric characteristics, as well as her nutritional status and diet, are of key importance. Breast milk minerals have been extensively studied with highly uneven results. In this work, a comparison will made with data across the world. To understand the factors that might explain the disparity, several minerals (Na, K, Ca, P, Mg, Fe, Se and I) have been analyzed using ICP-MS in a set of human milk samples (*n* = 75). The samples had an identical geographical origin (Galicia, in northwestern Spain) but different lactation circumstances, including maternal anthropometric data, lactating time, newborn sex and maternal adherence to healthy dietary patterns (Mediterranean Diet, MD, or Atlantic Diet, AD). The required concentrations of essential elements reported in the literature are similar to those found in these Spanish women. A univariate approach revealed that factors such as lactating time, body mass index (BMI) and newborn sex have a significant influence in breastmilk mineral content. According to multivariate linear regression analysis, minerals in milk are particularly associated with lactating time, but also with newborn sex, maternal BMI, age and diet pattern in some cases. More precisely, these results suggest that the iron and selenium concentrations in the milk of Galician donors may be positively influenced by maternal adherence to AD and MD, respectively.

## 1. Introduction

Maternal eating habits are important during lactation. In this period, the child needs energy and fundamental nutrients, not only to maintain life, but also to achieve rapid growth and the development of his tissues, organs and functions [1].

The Lancet series on Breastfeeding [2] and the publication Nurturing the Health and Wealth of Nations: The Investment Case for Breastfeeding [3] present some of the latest efforts to disseminate the almost indisputable scientific evidence supporting the benefits of breastfeeding for infants, for the mother, and even to lay the foundations for healthier and more prosperous societies.

During breastfeeding, maternal diet determines not only the mother’s nutritional status and, therefore, her health, but also that of the child. One of the latest systematic reviews [4] includes a wide range of studies on a broad spectrum of foods which can vary the nutritional characteristics of maternal milk. The main conclusion of this work is that the information available on this subject is scarce and diversified. This means that most of the evidence currently used in clinical practice to make recommendations is limited to studies that report only indirect associations. Therefore, further studies on the role of maternal diet in milk composition are needed.

The Mediterranean Diet (MD) is a dietary pattern that includes a high intake of olive oil, fruit, nuts, vegetables, legumes, moderate fish intake, poultry, red meat, wine and dairy products. It also features low consumption of processed meats and refined sugars. MD is intimately linked to the Mediterranean lifestyle, including Spain [5]. Many studies have been published about the health benefits of adherence to the MD pattern [6,7,8]. This style of diet could have several advantages during lactation, related to heart-healthy fatty acids and iron profile, along with dietary antioxidant factors and the low oxidative load of the foods consumed [9].

The dietary pattern of Northern Portugal and the Galicia region (northwest Spain) shares common characteristics with the Mediterranean Diet, but certain particularities suggest a typical Atlantic pattern that has been called the Southern European Atlantic Diet (SEAD) or Atlantic Diet (AD) [10]. The consumption of fish three/four times a week, fruits and vegetables, milk and dairy products, the moderate consumption of meat and the simplicity in the preparation of food, to maintain the quality of the raw materials and, therefore, the nutritional value, are characteristics of this type of diet. This style of eating could have many health advantages related to its supply of omega-3 fatty acids, vitamin D and other vitamins, Ca, Fe and other minerals [11,12,13].

In this work, the mineral content (Na, K, Ca, P, Mg, Fe, Se and I) was determined in a set of human milk samples donated by Spanish mothers living in the northwest of Spain. Data on maternal anthropometric characteristics, lactating time, adherence to healthy dietary patterns, i.e., MD or AD, and newborn sex were also collected and analyzed in order to determine the influence of these factors on the mineral content of breast milk from an identical geographical origin.

## 2. Materials and Methods

### 2.1. Samples and Data Collection

This work belongs to an observational cross-sectional study designed to investigate the breast milk composition of lactating mothers living in the north-west of Spain (Galicia). This region was chosen for the characterization of breast milk mineral composition on the basis of its proximity to the research group and also the coexistence of two healthy dietary patterns in a relatively small area. Recruitment, milk sampling and data collection were done by applying convenience sampling in collaboration with midwifery service and a local breastfeeding association. The study was registered in ClinicalTrials.gov with the identification number NCT03245697. This study was approved by the Galician Clinical Research Ethics Committee (Approval code 2016/280) and adhered to the principles of the Helsinki Declaration of 1975, as revised in 1983. Written informed consent was obtained from all participants.

Milk samples were donated by 85 lactating women. The mothers were interviewed and prescreened for the following exclusion criteria: mothers with acute or chronic diseases, suffering from any metabolic disorders, gestation of less than 36 weeks, abusers of drugs or alcohol. All babies were healthy and growing well. For the study, 75 of those mothers were selected. These nursing women were surveyed about personal data: age, medicines, tobacco use, anthropometric data (body weight, height and BMI), childbirth (date of delivery, natural or C-section and sex) and lactation (lactating time as months after delivery). Additionally, mothers were asked about their dietary habits to determine the degree of adherence to the MD or AD. A short questionnaire of 14 items (Mediterranean Diet Adherence Screener, MEDAS), validated for the Spanish population by the Mediterranean Diet Prevention group (PREDIMED), was used [14,15]. To get the score, each positive response relative to the MD was assigned a value of +1, or 0 when the question received a negative response. The sum of the values obtained for the 14-item questionnaire was used to appraise the MD adherence of participants [15]. Also, a short, 9-item questionnaire (SEAD) was used to determine the level of adherence to an Atlantic diet [12,16]. Human milk samples were collected at home by the mothers. Approximately 25 mL milk was extracted via manual expression or breast pump, before breastfeeding the child, and collected into a sterile plastic tube. The samples were stored at 3 to 5 °C and delivered to the laboratory within 12 h. All samples were collected before the first morning breastfeeding or two hours after the last session, when the child breastfeeds at night.

### 2.2. ICP-MS Method

Analytical-grade reagents and chemicals were used in the preparation of all solutions. Milk samples (5 mL) were digested with 8 mL HNO_3_ 69% (Hiperpur, Panreac) and 2 mL 33% H_2_O_2_ (Panreac) using the microwave digestion method (Milestone, Ethos1 Plus) (190 °C for 15 min at 1000 W). Once digested, the resulting solutions were cooled and diluted to a final volume of 50 mL with H_2_O (Milli-Q). Determination of mineral contents (Na, K, Ca, P, Mg, Fe and Se) was carried out by inductively coupled plasma-mass spectrometry (ICP-MS) (Agilent 7700×, with a sample introduction system formed by a Micromist glass low-flow nebulizer, a double-pass glass spray chamber with Peltier system (2aC) and quartz torch. The operating parameters are given in Table 1.

Digestion and determination of iodine in the milk samples was also done by ICP-MS following the protocol European Standard EN 15111 [17]. In both methods, two blanks and a certified reference material (Reference material 1549 Not-Fat milk power, NIST) were included in each digestion batch. Working standard solutions were prepared by dilution of stock standard solutions (Panreac) to the desired concentration in NO_3_H-H_2_O in the same proportion as the samples. Calibration curves ranges (5 points) were selected to match the expected concentrations for all elements in milk samples. The correlation coefficients for each element were equal to or greater than 0.9999.

### 2.3. Statistics

A data analysis was performed using SPSS version 24 statistical computer software (SPSS, Chicago, IL, USA). All data were assessed for normality using a Kolmogorov-Smirnov test, and for equality of variances using the Levene’s test. Accordingly, a Student’s *t* test or Mann-Whitney U test was used to detect differences between the two independent groups of samples constructed on the basis of cut-off values for lactating time, BMI and newborn sex. For all tests, *p* < 0.05 was considered significant.

Further, to assess associations between minerals and maternal factors, multivariate linear regression analysis was used (stepwise method), where mineral levels were the dependent variables, and the dietary scores (MD or AD) and maternal characteristics (lactating time, BMI, maternal age, and newborn sex) were considered as independent variables. Variables that did not show a normal distribution were log-transformed (natural logarithms). The final models were further evaluated with the aid of their respective normal probability plots of residuals. Two-sided *p* values < 0.05 were considered statistically significant.

## 3. Results and Discussion

### 3.1. Maternal Characteristics

Among the 85 initial donors, 75 were selected for this study, all of whom were healthy, nursing mothers from the northwest of Spain. Three donors said they were smokers at the time of sampling. Only 9 women (12%) underwent a C-section delivery and 60% were primiparous. Significant data on gestation and on maternal and newborn characteristics are provided in Table 2. Continuous data are expressed as mean and standard deviation (SD), and categorical variables are expressed as percentages.

### 3.2. Galician Human Milk Composition Compared with Worldwide

In recent years, the number of publications on the mineral composition of breast milk has increased. Many of these studies have been conducted in different populations with very different lifestyles and diets. It is possible that these variables influence the disparate results found in the literature.

The next three tables present the mineral concentrations in mature human milk collected in different countries, according to the scientific literature, with each table containing representative minerals from three categories [18]: (1) minerals such as calcium, phosphorus and magnesium, that are regulated by homeostatic mechanisms in maternal serum (Table 3); (2) electrolytes, including sodium and potassium, which are determined by a gradient of electrical potential in the secreting cell (Table 4); and (3) trace elements, such as iron, selenium or iodine, which exist in very low concentrations in human milk (Table 5). Data since 1980 have been included, and works have been selected from populations in different parts of the world, including Spain. The table also reflects the concentrations obtained for these minerals in the breast milk of Galician women analyzed in this study.

Calcium is the second most abundant mineral in breast milk [18]. EFSA expert opinion [42] considers that the average calcium intake suitable for most babies in the first half year of life is 200 mg per day. This could be achieved with an intake of 800 mL of milk per day, if that milk has a calcium concentration of 250 mg/L. In 2017, a comparison was published in PLoS ONE [36] between countries with varying degrees of development (Argentina, Namibia, Poland, and the United States). The results, in terms of calcium concentration, showed a high degree of variability among the different populations. Calcium concentrations were significantly lower in the Namibian population than elsewhere, i.e., while the mean calcium concentration in U.S. breast milk was 278.62 mg/L, in Namibia, it was 143.83 [36,39]. These results are consistent with previous data, in which calcium was found to be one of most variable essential elements among different populations (Nigeria 186 [32]; Gambia 208.93 [28]; Brazil 250 [31]; Japan, 250 [30]; Sweden 305 mg/L [34]) [39]. The authors of reported similar calcium concentrations in New Zealand, depending on ethnicity, i.e., ranging from 275 to 309 mg/L. In the present study, the average calcium concentration was 279.99 mg/L, very close to the countries with economies similar to that of Spain, and higher than the levels reported for countries with lower levels of development.

Low phosphorus breast milk concentrations have been associated with improved calcium absorption and a number of other benefits for the newborn [43]. Data in different areas of the world show less disparity than those obtained for calcium, which is in line with the observations of Björklund et al. [34], who referred to a fine regulation of the P content in breast milk. Only Sabatier et al. [40] found values in some samples below 100 and above 200 mg/L, although the mean was 148 mg/L. The same observation was made in this work, i.e., the phosphorus concentration ranged from 73.00 to 219.57, with a mean of 126.46 mg/L. The average Ca/P ratio was 2.29.

Magnesium is the second most abundant intracellular cation after sodium. Its concentrations in breast milk are regulated by homeostatic mechanisms which ensure magnesium stability, regardless of constitutional, metabolic or dietary variables. The concentrations of this essential element reported in the literature (see Table 2) are very similar among all populations. The values ranged in all cases between 10 and 50 mg/L, although in most studies, the concentrations were close to 30. In this study, the average concentration among Galician mothers was 33.22. In an EFSA report [42], the mean was 31 mg/L. The panel considers that, assuming an intake of 800 mL of milk per day, an average magnesium concentration of 31 mg/L would provide 25 mg of that mineral per day, which is suitable for most babies during the first half year of life.

Electrolyte concentrations (sodium, potassium and chlorine) in human milk are determined by the gradient of electrical potential in the secreting cell rather than by maternal nutritional status, being lower in mature milk than in calostrum [46]. Some researchers have reported increases in sodium levels, and occasionally chloride, of 5 to 40 times in human milk associated with emotional stress, mastitis and decreased milk production [47]. In a WHO/IAEA report [26], sodium values were between 60 and 170 mg/L, while for EFSA [42], values ranged from 140 to 160 mg/L. In the literature, values were higher than 200 mg/L [19,31,34,37,40]. The authors observed an average of 176.94, albeit with wide variability. The daily intake of 150 mg (achieved for example with a daily intake of 800 mL of 120 mg/L sodium concentration milk) is appropriate for most babies in the first year of life [42].

Regarding potassium, the WHO/IAEA [26] showed differences among mothers and among countries. Samples from Hungary and Sweden had very high values, while samples from Nigeria showed quite low levels. In this review (see Table 2), only samples described by Dewey et al. [24] and Perrin et al. [38] had averages below 400 mg/L. EFSA [42] considers that the appropriate intake for most children up to 6 months of age is 400 mg per day, i.e., with a daily consumption of 800 mL of breast milk, a concentration of 500 mg/L would be required. The average for Galician mothers was 456.62 mg/L.

EFSA [42] considers that the mean iron intake from breast milk (0.3 mg per day for a concentration of 0.35 mg/L and an intake of 800 mL/day) is generally sufficient to ensure that the iron status in the first half year of life remains within a healthy range [65]. However, it should be noted that for formula-fed infants and some infants after 4 to 6 months of age, an intake equivalent to this value is not sufficient to keep the iron status within the normal range. The iron concentrations observed in many studies (including the present one, 0.23 mg/L) were below 0.35 mg/L. Nevertheless, the absorption of iron from breast milk is somehow greater than its absorption from infant formula or complementary foods fortified with iron [66]. In addition, babies have been shown to have the ability to regulate iron absorption when iron reserves decrease [50,67] this ability makes babies more resistant to iron deficiency than a simple factorial approach would predict [42].

Breastfed infants rely on breast milk iodine to meet their physiological needs, which, in turn, depends on the mother’s iodine status, as well as on other factors [68]. According to the EFSA [42], the concentration of iodine in the mature breast milk of European women is in the range of 50 to 100 µg/L. Assuming a milk intake of 0.8 L per day, between 40 and 80 µg of iodine per day would be consumed. The panel considers that an iodine intake of 90 µg per day is suitable for most children in the first half year of life. In our review, the levels of iodine in most populations were not taken into account, with results only being reported from countries with high standards of living and other developing countries. The concentrations of iodine ranged from 49 µg/L in Swiss women [63] to 144 µg/L in Spanish women, as sampled by Fernández Sánchez [59]. In the present study, the mean value was 95.44 µg/L.

Globally, there are large differences in soil selenium content, and therefore, in food availability. A wide range of selenium concentrations has been observed in human milk, depending on the amount of selenium consumed by the mother from natural foods. The concentrations obtained differ from one country to another [69]. The WHO/IAEA [26] reported average values ranging from 13 to 33 µg/L. Samples from the Philippines had very high values, while in Sweden, values were relatively low. According to EFSA [42], selenium concentrations in the breast milk of European women ranged from 3 to 84 µg/L, with an average value of 16.3 µg/L. The panel considers that 12.5 µg average intake per day of Selenium in breast milk (i.e., 16 µg/L × 0.8 L) is suitable for most infants in the first half year of life. In this review, the highest concentrations of selenium were found in samples from Turkey [58]. In Galician women, an average concentration of 15.13 µg/L was observed with wide variability, i.e., a little lower than what the EFSA [43] considers optimum.

### 3.3. Influential Factors on Breast Milk Mineral Content

Milk production is a complex process conditioned by maternal and child factors. The mineral content is quite variable, and the study of influencing factors is very important for lactation management and to provide adequate dietary advice. In order to establish the possible relationship of categorical factors with the mineral content of breast milk, univariate statistical analyses were performed. Samples were divided into two groups for each category: less and more than 6 months of continued breastfeeding; BMI greater than or equal to 25 kg/m^2^; and infant gender. The sodium, potassium, calcium, magnesium, phosphorus, iron, selenium and iodine contents across factor categories and significant differences are presented in Table 6.

Samples were divided into two groups according to lactation time (LT) because, at 6 months, babies normally start complementary feeding. Only the Mg and K content in both groups of mothers, according to LT, followed a normal distribution. There aren’t significant differences when lactation is prolonged more than 6 months. The results obtained for Mg in the sampled women are homogeneous, in line with Dórea [70], who refers to the homeostatic regulation of Mg to explain the limited variability in the concentration of magnesium in breast milk. With regard to potassium, although different values have been found in different parts of the world, among Galician women, there was no difference during lactation, whether it is more or less prolonged. A nonparametric Mann-Whitney U test was used for the other minerals. Using this statistical approach, significant differences between early and late lactation (≥6 months) were found for Ca, Ca/P ratio and I content. Calcium and phosphorus are major minerals that are tightly regulated in maternal serum. In milk, calcium is linked to casein and citrate, or occurs as ionic calcium; the concentration of the latter is more stable, which suggests a homeostasis similar to that of calcemia [71]. The findings in this work were consistent with those of other studies that observed a gradual decline of calcium in maternal milk with the advance of maternal lactation [38,72,73]. No significant differences in phosphorus content were found throughout lactation. This could be explained by the fine regulation of P content in breast milk [34]. Therefore, the differences found in the Ca/P ratio (i.e., declining in milk after 6 months of lactation) are due to the decrease of calcium. Feeley et al. [56] observed significant differences in Fe content with lactating time. As in this work, other authors such as Arnaud et al. [74] or Emmett and Rogers [75] reported, however, a constant level of iron throughout lactation. The iodine content in breast milk showed significant differences between the samples from less than six months and those with longer lactation times. Dror and Allen [76] also saw a gradual decline in iron levels in mature milk across longitudinal studies.

Concerning maternal anthropometric characteristics (BMI), there are significant differences in Na levels in breast milk, with higher concentrations in women with BMIs greater than or equal to 25. This fact can potentially be explained by taking into account a possible failure in prolonged lactation (more than 12 months) in 7 women with BMIs greater than 25. Sodium content increases have been repeatedly linked to lactation failure [77,78,79]. Examining the sex of newborns, significant differences were found in the mineral content of breast milk. There were differences in Ca/P levels, with higher ratios in mothers who had given birth to girls. Also, there were significant differences in Na and P contents. Mothers of girls showed higher levels of sodium, while mother of boys showed higher levels of P. Although differences have been found [80], the literature does not establish relationships between newborn sex and mineral content [74].

#### Multivariate Approach

Multiple linear regression analysis was used to control the data for confounding effects by modelling mineral levels (dependent variables) using lactating time, BMI, maternal age, newborn sex and dietary scores (MD or AD) as independent variables. It was not possible to construct a model for P or I with any of the independent variables. The contents of these minerals cannot be explained by any of the variables studied, perhaps due to their homeostasis throughout lactation, independent of the factors studied in this work. The regression models are presented in Table 7.

Lactating time was found to be an important factor, showing a positive correlation with Na concentration, in agreement with mean values reported in Table 6. This fact was not significant in the univariate approach, probably due to the high data dispersion in women lactating for more than 6 months (133.53 ± 55.16 vs. 256.37 ± 242.31). Sodium increases in prolonged lactations have also been demonstrated by other authors [77,78,79]. Similar considerations can be made with regard to K, i.e., lactating time was negatively associated with K concentrations. However, these differences were not significant when dividing the data into categories, nor were they visually evident. A gradual decline in calcium and the Ca/P ratio with the advance of maternal lactation was statistically significant, as reported in Table 6. Additionally, lactating time was shown to be negatively correlated with Ca and Ca/P values in multivariate regression, in accordance with the literature [32,72,73]. Maternal characteristics (BMI) and newborn sex were positively associated with Na concentrations in breast milk, as was shown in our univariate analysis.

In their systematic review on the quantitative relationships between maternal diet and the composition of breast milk, Bravi et al. [4] emphasized the dispersion and weak evidence that exists to support a certain type of dietary pattern among nursing women. A large number of observational studies have been published about the relationship between the consumption of certain foods and the mineral content of human milk. As examples, rice consumption has been linked to the content of Zn [81], the total energy consumed by mothers with the content of Fe and Zn [82], and the consumption of eggs or vegetarianism with the content in Se [83,84]. Today, the study of the effects of nutrition on health has changed the focus from isolated nutrients to dietary patterns [85]. This attempt to relate maternal healthy dietary patterns with the content in milk of representative minerals is the main strength of our study. There is strong evidence for the positive health effects of a MD, as well as for the dietary pattern of northern Portugal and the Galicia region (northwest Spain), i.e., the Atlantic Diet [5,6,7,14,15,16]. Associations between mineral breastmilk content and healthy dietary patterns were assessed by multiple linear regression analysis, considering AD and MD scores as independent variables in the different models.

The best resulting significant model (*p* < 0.05) for Fe concentration in breast milk included only the AD score as an independent variable. AD adherence showed a positive association with Fe content in breast milk. This dietary pattern is characterized by moderate consumption of meat [11,12,13], and the energy consumed in each meal is usually high. However, according to the literature, maternal dietary iron has a little effect on milk iron concentration [86]. In this work, Fe was shown to be in the range of 0.06 to 0.75 mg/L. Some of the samples were below 0.30, i.e., the recommended value [13]. Perhaps maternal adherence to an AD could improve the Fe content in breastmilk, which is so important in the child’s development. Galician breastmilk showed a wide range of Se concentrations (4.37 to 148.97 µg/L). MD adherence showed a positive correlation with Se concentration in breast milk. Most MD-adherent women consume more vegetables, nuts and legumes in their diets, all of which could enrich breast milk [83,87].

## 4. Conclusions

In this work, a comparison was made with data across the world and samples from identical geographical origin but different lactation circumstances. The study found that the concentrations of Na, K, Ca, Mg, Fe, Se and I in breast milk present significant variability. However, if we suppose that exclusively breastfed babies consume per day 800 mL of milk, the total intake of these minerals is sufficient in most cases. Using a statistical approach for levels, a significant decline between early and late lactation (≥6 months) was found for Ca, the Ca/P ratio and I content. Women with high BMIs had higher concentration in Na levels, and higher Na and Ca/P ratios were found in mothers who had given birth to girls, while mothers of boys had higher levels of P. The results obtained with the multiple regression approach confirmed that a gradual decrease in Ca and Ca/P occurs throughout lactation. Therefore, continued breastfeeding could lead to Ca deficits. Complementary feeding after the age of 6 months would compensate for this. Our multivariate approach also revealed an increase in Na and a decrease in K contents as lactation progresses. This statistical approach suggested a relationship between healthy AD and MD dietary patterns and higher concentrations of Fe and Se, respectively, in maternal milk. More robust studies are necessary to determine whether these dietary patterns may further influence the breast milk mineral content and help develop appropriate interventions to ensure mother and child health. Future research should aim to assess the influence of the diet of lactating mothers on the mineral content of their milk.

## Figures and Tables

**Table 1:** ICP-MS parameters setting.

Plasma Parameters		Ion Lenses (V)		Octopole Parameters (V)	
RF Power (W)	1550	Extract 1	0	OctP RF	180
Sample Depth	8	Extract 2	−175	OctP Bias	−18
Carrier Gas (L/min)	1.1	Omega Bias	−100	**Reaction Cell (mL/min)**	
Nebulizer Pump (rps)	0.1	Omega Lens	12.6	He Gas	3.6
S/C Temp (°C)	2	Cell Entrance	−40	**Detector Parameters**	
		Cell Exit	−60	Discriminator (mV)	4.5
		Deflect	0.4	Analog HV (V)	1730
		Plate Bias	−60	Pulse HV (V)	954
		QP Bias	−15		

**Table 2:** Pregnancy and maternal characteristics, continuous and categorical variables (*n* = 75).

Maternal Data	Mean	Median	SD	Min	Max
**Pregnancy Time (weeks)**	39.91	40.00	1.29	36.00	42.29
**Maternal Age (years)**	35.50	35.00	3.99	27.00	46.00
**Maternal Height (m)**	1.65	1.65	0.05	1.53	1.77
**Maternal BMI (kg/m^2^)**	24.11	23.34	3.88	17.99	35.03
**Lactating time (months)**	8.02	3.72	10.43	0.77	58.97
**MD adherence (score)**	9.00	8.00	2.26	2.00	12.00
**AD adherence (score)**	3.87	4.00	1.52	0.00	7.00
**Infant gender: *n* ♂ (%)/*n* ♀ (%)**	33 (44.00)/42 (56.00)		**C-section delivery (%)**		12.00
**Time lactation < 6 months (%)**	64.00		**Parity number 1st child (%)**		60.00

**Table 3:** Mineral regulated by homeostatic mechanisms [18] reported in mature human milk in different populations.

Mineral	Authors, Year, Reference, Country	Mean	SD	Range	Units
**Calcium**					
	Gross et al., 1980, [19], USA	249.8			mg/L
	Fransson & Lonnerdal, 1983, [20], USA	279	28.6		mg/L
	Dewey & Lonnerdal, 1983, [21], USA	253.0			mg/L
	Garza et al., 1983, [22], USA	213.4			mg/L
	Feeley et al., 1983, [23], USA	262.0			mg/L
	Dewey et al., 1984, [24], USA	229.4	40.1		mg/L
	Butte et al., 1984, [25], USA	154.9			mg/L
	WHO & IAEA, 1989, [26]			220.0–300.0	mg/L
	Allen et al., 1991, [27], USA	280.7	20.1		mg/L
	Dorea et al., 1999, [28], Gambia	208.93	24		mg/L
	Friel et al., 1999, [29], USA	279.6			mg/L
	Yamawaki et al., 2005, [30], Japan	249.0	16.52		mg/L
	Mastroeni et al., 2006, [31], Brazil	250	31.0		mg/L
	Thacher et al., 2006, [32], Nigeria	186	41		mg/L
	Shi et al., 2011, [33], China	334.00	70.00		mg/L
	Björklund et al., 2012, [34], Sweden	305.00	45.00		mg/L
	Andrade et al., 2104, [35] Brazil	142.30	21.60		mg/L
	Klein et al., 2017, [36], USA	268.7	59.34		mg/L
	Klein et al., 2017, [36], Namibia	143.83	64.67		mg/L
	Klein et al., 2017, [36], Poland	227.06	36.72		mg/L
	Klein et al., 2017, [36], Argentina	231.79	37.47		mg/L
	Aumeistere et al., 2017, [37], Latvia			227.52–398.34	mg/L
	Perrin et al., 2017, [38], USA	194.0			mg/L
	Butts et al., 2018, [39], New Zealand			275–309	mg/L
	Sabatier et al., 2019, [40], Switzerland	286	47		mg/L
	Daniels et al., 2019, [41], Indonesia			247–300	mg/L
	Present study, 2019, NW Spain	279.99		136.44–463.26	mg/L
**Phosphorus**					
	Gross et al., 1980, [19], USA	150.8			mg/L
	Feeley et al., 1983, [23], USA	133.0			mg/L
	Butte et al., 1984, [25], USA	144.4			mg/L
	Mastroeni et al., 2006, [31], Brazil	137.0	20.0		mg/L
	Thacher et al., 2006, [32], Nigeria	126.81	32.99		mg/L
	Sabatier et al., 2019, [40], Switzerland	148	30		mg/L
	Daniels et al., 2019, [41], Indonesia			119–145	mg/L
	Present study, 2019, NW Spain	126.46		73.00–219.57	mg/L
**Magnesium**					
	Gross et al., 1980, [19], USA	26.2			mg/L
	Fransson & Lonnerdal, 1983, [20], USA	35.04	2.46		mg/L
	Dewey & Lonnerdal, 1983, [21] USA	31.7			mg/L
	Feeley et al., 1983, [23], USA	50			mg/L
	Dewey et al., 1984, [24], USA	31.2	5.6		mg/L
	Butte et al., 1984, [25] USA	34.6			mg/L
	WHO & IAEA, 1989, [26]			29.00–38.00	mg/L
	Allen et al., 1991, [27], USA	38.7	2.4		mg/L
	Friel et al., 1999, [29], USA	29.1			mg/L
	Yamawaki et al., 2005, [30], Japan	28.33	4.16		mg/L
	Mastroeni et al., 2006, [31], Brazil	29.9	5.00		mg/L
	Shi et al., 2011, [33], China	37.00	10.00		mg/L
	Björklund et al., 2012, [34], Sweden	28.00	4.80		mg/L
	Andrade et al., 2104, [35], Brazil	39.80	4.20		mg/L
	Aumeistere et al., 2017, [37], Latvia			25.73–49.52	mg/L
	Butts et al., 2018, [39], New Zealand			10.2–30.8	mg/L
	Sabatier et al., 2019, [40], Switzerland	32	7		mg/L
	Daniels et al., 2019, [41], Indonesia			26.3–35.9	mg/L
	Present study, 2019, NW Spain	33.22		19.86–50.83	mg/L

**Table 4:** Electrolyte concentration [18] reported in mature human milk in different populations.

Electrolyte	Authors, Year, Reference, Country	Mean	SD	Range	Units
**Sodium**					
	Gross et al., 1980, [19], USA	215.3			mg/L
	Dewey & Lonnerdal, 1983, [21], USA	185.9			mg/L
	Garza et al., 1983, [22], USA	131.9			mg/L
	Dewey et al., 1984, [24], USA	94.6	51.4		mg/L
	Butte et al., 1984, [25], USA	154.9			mg/L
	WHO & IAEA, 1989, [26]			90.0–130.0	mg/L
	Allen et al., 1991, [27], USA	154.8	28.50		mg/L
	Wack et al., 1997, [44], USA			144.5–124.5	mg/L
	Yamawaki et al., 2005, [30], Japan	120.67	16.50		mg/L
	Mastroeni et al., 2006, [31], Brazil	205.0	156.00		mg/L
	Björklund et al., 2012, [34], Sweden	217.00	77.00		mg/L
	Roy et al., 2014, [45], India	527.40	199.00		mg/L
	Aumeistere et al., 2017, [37], Latvia			58.56–256.38	mg/L
	Perrin et al., 2017, [38], USA	67.9			mg/L
	Sabatier et al., 2019, [40], Switzerland	235.0	237		mg/L
	Daniels et al., 2019, [40], Indonesia			101–193	mg/L
	Present study, 2019, NW Spain	176.94		44.75–942.67	mg/L
**Potasium**					
	Gross et al., 1980, [19], USA	582.5			mg/L
	Dewey & Lonnerdal, 1983, [21], USA	457.2			mg/L
	Dewey et al., 1984, [24], USA	397.7	71.0		mg/L
	WHO & IAEA, 1989 [26]			410.0–550.0	mg/L
	Allen et al., 1991 [27], USA	542.9	48.2		mg/L
	Wack et al., 1997, [44], USA			504.4–448.8	mg/L
	Yamawaki et al., 2005, [30], Japan	437.33	7.57		mg/L
	Mastroeni et al., 2006, [31], Brazil	462	84		mg/L
	Shi et al., 2011, [33], China	540.00	146		mg/L
	Björklund et al., 2012, [34], Sweden	633.00	40		mg/L
	Aumeistere et al., 2017, [37], Latvia			445.33–736.71	mg/L
	Perrin et al., 2017, [38], USA	363.7			mg/L
	Sabatier et al., 2019, [40], Switzerland	575	92		mg/L
	Daniels et al., 2019, [41], Indonesia			402–499	mg/L
	Present study, 2019, NW Spain	456.62		342.98–622.25	mg/L

**Table 5:** Trace element concentration [18] reported in mature human milk in different populations.

Mineral	Authors, Year, Reference, Country	Mean	SD	Range	Units
**Iron**					
	Fransson & Lonnerdal, 1983, [20], USA	0.36	0.19		mg/L
	Dewey & Lonnerdal, 1983, [21], USA	0.2			mg/L
	Garza et al., 1983, [22], USA	0.3			mg/L
	Feeley et al., 1983, [48], USA	0.76			mg/L
	Dewey et al., 1984, [24], USA	0.2	0.1		mg/L
	Gunshin et al., 1985, [49], Japan	0.32	0.16		mg/L
	WHO & IAEA, 1989 [26]			0.07–0.35	mg/L
	Domellöf et al., 2004, [50], Honduras	0.29	0.21		mg/L
	Yamawaki et al., 2005, [30], Japan	1.19	2.51		mg/L
	Mastroeni et al., 2006, [31], Brazil	0.90	0.5		mg/L
	Hannan et al., 2009, [51], USA	0.5	1.0		mg/L
	Shi et al., 2011, [33], China	0.50	0.2		mg/L
	Mello-Neto et al., 2012, [52], Brazil	0.3	0.2		mg/L
	Björklund et al., 2012, [34], Sweden	0.33	0.19		mg/L
	Roy et al., 2014, [45], India	0.162	0.06		mg/L
	Andrade et al., 2104, [35], Brazil	0.27	0.4		mg/L
	Klein et al., 2017, [36], USA	1.27	0.26		mg/L
	Klein et al., 2017, [36], Namibia	1.53	0.86		mg/L
	Klein et al., 2017, [36] Poland	1	0.15		mg/L
	Klein et al., 2017, [36], Argentina	0.99	0.21		mg/L
	Aumeistere et al., 2017, [37], Latvia	0.31			mg/L
	Perrin et al., 2017, [38], USA	0.20			mg/L
	Sabatier et al., 2019, [40], Switzerland	0.44	0.26		mg/L
	Daniels et al., 2019, [41], Indonesia	0.33			mg/L
	Peixoto et al., 2019, [53] Brazil			0.01–0.52	mg/L
	Present study, 2019, NW Spain	0.23		0.06–0.75	mg/L
**Selenium**					
	WHO & IAEA, 1989 [26]			13.0–33.0	µg/L
	Casey et al., 1989, [54], USA			7–20.0	µg/L
	Krachler et al., 1998, [55], Austria	17			µg/L
	Zachara & Pilecki, 2000, [56], Poland			8.81–11.58	µg/L
	Navarro-Blasco & Alvarez-Galindo, 2004, [57], Spain	16.3	4.7		µg/L
	Yamawaki et al., 2005, [30], Japan	15.3	2.5		µg/L
	Özdemir et al., 2008, [58], Turkey	68.63	7.78		µg/L
	Hannan et al., 2009, [51], USA	15.9	4.1		µg/L
	Shi et al., 2011, [33], China	15.0	6.0		µg/L
	Björklund et al., 2012, [34], Sweden	13.00	2.6		µg/L
	Butts et al., 2018, [39], New Zealand			13–16	µg/L
	Sabatier et al., 2019, [40], Switzerland	15.0	4.2		µg/L
	Daniels et al., 2019, [41], Indonesia			8.7–12.9	µg/L
	Peixoto et al., 2019, [53] Brazil			2.5–70.6	µg/L
	Present study, 2019, NW Spain	15.13		4.37–148.97	µg/L
**Iodine**					
	Fernández Sánchez et al., 2007, [59], Spain	144	93.2		µg/L
	Dasgupta et al., 2008, [60], USA	110			µg/L
	Leung et al., 2009, [61], USA	51.4			µg/L
	Hannan et al., 2009, [51], USA	47.8	17.1		µg/L
	Andersson et al., 2010, [62], Switzerland	49			µg/L
	Andersen et al., 2014, [63], Denmark	83			µg/L
	Mekrungcharas et al., 2014, [64], Thailand	129.7			µg/L
	Sabatier et al., 2019, [40] Switzerland	87	41		µg/L
	Present study, 2019, NW Spain	95.44		11.25–247.63	µg/L

**Table 6:** Sodium, potassium, calcium, magnesium, phosphorus, iron, selenium and iodine content in breast milk, as well Ca/P ratio, presented as mean, standard deviation and range across different groups of samples (lactating time or LT, BMI, newborn sex).

Mineral	Statistic	LT < 6 Months (*n* = 48)	LT ≥ 6 Months (*n* = 27)	BMI < 25 kg/m^2^ (*n* = 41)	BMI ≥ 25 kg/m^2^ (*n* = 34)	Infant Gender ♂ (*n* = 33)	Infant Gender ♀ (*n* = 42)
Na (mg/L)	Mean	133.53	256.37	132.37 *	205 *	212.39 *	131.30 *
	SD	55.16	242.31	66.72	156.08	200.60	30
	Range	44.75–303.88	46.81–942.67	44.75–371.21	69.71–942.67	44.75–389.21	68.04–942.67
K (mg/L)	Mean	465.37	438.03	449.00	476.86	450.42	463.60
	SD	64.57	60.29	58.49	77.01	65.54	282.96
	Range	345.57–622.25	342.98–550.27	345.57–579.69	342.98–622.25	353.34–622.25	342.98–599.71
Ca (mg/L)	Mean	295.15 *	251.94 *	281.93	275.09	282.96	278.69
	SD	55.18	58.24	59.72	56.90	63.94	55.28
	Range	196.1–463.26	136.44–383.5	192.60–463.26	136.44–382.50	197.81–381.38	136.44–463.26
P (mg/L)	Mean	126.79	127.02	133.38	120	122.38 *	132.66 *
	SD	26.56	34.10	29.47	30.07	32.35	23.44
	Range	79.82–177.95	73–219.57	83.93–210.57	73.00–177.95	82.50–177.95	73–219.57
Ca/P (ratio)	Mean	2.39 *	2.07 *	2.17	2.39	2.41 *	2.13 *
	SD	0.55	0.54	0.55	0.60	0.65	0.42
	Range	1.34–4.44	1.16–3.36	1.29–4.44	1.16–3.44	1.29–3.14	1.16–4.44
Mg (mg/L)	Mean	32.96	33.63	33.38	33.20	34.56	31.78
	SD	6.82	5.75	7.08	4.85	6.43	6.28
	Range	19.86–50.83	20.77–44.78	19.86–50.83	20.77–44.78	19.86–44.78	20.77–50.83
Fe (mg/L)	Mean	0.22	0.24	0.22	0.24	0.21	0.24
	SD	0.10	0.17	0.09	0.15	0.10	0.14
	Range	0.10–0.45	0.06–0.75	0.06–0.42	0.06–0.75	0.06–0.45	0.06–0.74
Se (µg/L)	Mean	11.41	21.99	11.60	12.07	18.95	10.66
	SD	5.00	33.55	5.65	4.85	27.44	4.21
	Range	4.37–25.85	6.18–148.97	4.37–28.13	5.30–148.97	4.37–21.14	6.18–148.97
I (µg/L)	Mean	110.92 *	75.27 *	104.83	97	96.03	99.16
	SD	53.61	61.61	62.09	53.36	68.01	52.72
	Range	26.71–233.19	11.25–247.63	11.25–247.63	26.71–233.19	26,71–247.63	11.25–233.19

Significant differences between groups (*).

**Table 7:** Multiple regression analysis for modelling breast milk minerals.

Breast Milk Minerals (Dependent Variable)	Maternal Characteristics (Independent Variables)	ß	ß SE	ß C.I. (95%)	*p*	Adjusted R^2^	ANOVA for the Model (*p*)
**Na**	Constant	2.77	0.53	1.72–3.82	0.000	0.231	0.000
	Lactating time	0.20	0.06	0.09–0.31	0.001		
	Newborn sex	0.38	0.13	0.13–0.64	0.003		
	BMI	0.04	0.02	0.01–0.07	0.022		
**K**	Constant	402.49	62.70	277.50–527.49	0.000	0.170	0.000
	Lactating time	−25.59	6.66	−38.85–−12.32	0.000		
	Maternal age	5.11	1.84	1.44–8.77	0.007		
**Ca**	Constant	359.64	31.32	297.22–422.06	0.000	0.072	0.011
	Lactating time	−16.00	6.15	−28.26–−3.75	0.011		
**Ca/P**	Constant	3.17	0.33	2.52–3.83	0.000	0.099	0.006
	Lactating time	−0.18	0.06	−0.31–−0.06	0.006		
**Mg**	Constant	19.08	6.61	5.91–32.26	0.005	0.047	0.035
	Maternal age	0.40	0.19	0.03–0.77	0.035		
**Fe**	Constant	0.15	0.03	0.09–0.22	0.000	0.046	0.047
	AD score	0.05	0.02	0.00–0.10	0.047		
**Se**	Constant	−14.31	7.61	−29.52–0.90	0.065	0.148	0.005
	BMI	0.60	0.21	0.19–1.01	0.005		
	MD score	4.88	2.30	0.28–9.47	0.038		
	Newborn sex	3.01	1.48	0.06–5.97	0.046		
